# A Case of Coccidioides Meningitis in an Immunocompetent Host and the Challenges in Diagnosis

**DOI:** 10.1155/crdi/4277040

**Published:** 2025-10-21

**Authors:** Mayesha Sharaf, Li-Chien Chen

**Affiliations:** Internal Medicine, Mountain View Hospital, Las Vegas, Nevada, USA

**Keywords:** cerebrospinal fluid, coccidioides meningitis, dexamethasone, fluconazole, fungal meningitis, headache, syndrome of inappropriate antidiuretic hormone release

## Abstract

Coccidioides is the causative organism for Coccidioidomycosis or otherwise known as “Valley fever.” Valley fever usually presents as upper respiratory tract illness which can often be complicated by dissemination. Although immunosuppression increases the risk of disseminated disease, it can also be seen in immunocompetent patients. Coccidioides meningitis is the most feared outcome of disseminated coccidioides infection, rendering early diagnosis and treatment imperative. However, the diagnosis of coccidioides meningitis is challenging due to its vague presentation and its resemblance to other CNS disorders, both infectious and noninfectious. Moreover, serum and cerebrospinal fluid serologies, which are the mainstay in diagnosing coccidioides meningitis, can be inconclusive during the early phases of the disease process, potentially leading to missed diagnosis. Therefore, keeping a very high index of suspicion for coccidioides meningitis is crucial. Initiating antifungals early in suspected coccidioides meningitis even with inconclusive serologies may be appropriate as well. We present a case of coccidioides meningitis in an immunocompetent patient and the associated diagnostic challenges to guide physicians with the diagnosis and prevent fearsome complications.

## 1. Introduction

Coccidioides is a dimorphic fungus that is endemic to the hot, dry soils of southwestern United States including parts of California, Arizona, Nevada, New Mexico, Texas, Utah, parts of Mexico, and Central and South America. Coccidioides causes coccidioidomycosis, also known as Valley fever. It is primarily a lung infection, commonly acquired through inhalation of arthroconidia found in dust or soil. Infection can range from asymptomatic or mild respiratory illness to severe disseminated disease [1]. Meningitis is the most feared and devastating complication of Coccidioidal infection. Coccidioides meningitis is insidious and progressive, usually causes chronic meningitis, complicated by hydrocephalous or stroke [2].

Coccidioides meningitis is fatal if left untreated rendering early diagnosis imperative. However, central nervous system (CNS) involvement of Coccidioides usually presents with headache and confusion. Initial presentation can mimic other forms of CNS infection including bacterial or tubercular meningitis, noninfectious CNS conditions including intracranial bleeding, vasculitis or neoplasms, and even metabolic derangements including electrolyte abnormalities. Analysis of cerebrospinal fluid (CSF) in the setting of clinical suspicion is the mainstay of diagnosis. CSF is usually analyzed for opening pressure, cell count, glucose, protein, coccidioides serology via immunodiffusion (ID) and complement fixation (CF) and anti-coccidioides antibodies immunoglobulin G (IgG) and immunoglobulin M (IgM) via enzyme immunoassay (EIA). Coccidioides antigen testing in CSF has shown to be effective as well [3]. Whereas CSF opening pressure, cell count, glucose, and protein are usually readily available, CSF studies anti-coccidioides antibodies and coccidioides antigen detection can be time-consuming. Moreover, coccidioides antibodies may not be detectable during early disease phases. Often time, positive serum serology via ID or CF in appropriate clinical setting is the most common way of diagnosing coccidioides meningitis [4]. However, a negative serum serology does not definitively rule out coccidioides meningitis. Specially during the early disease process when the antibodies have not formed yet, making early diagnosis of coccidioides meningitis challenging. Given the fatality of the disease, a missed diagnosis can bring on significant harm to the patient.

Disseminated coccidioidomycosis is rare. It occurs in 1%–3% of cases, and pregnancy and immunosuppression are most commonly associated with disseminated coccidioidomycosis [5]. CNS involvement can occur in up to 50% of patients with disseminated coccidioidomycosis. Coccidioides meningitis has been reported in an immunocompromised host following recent coccidioidomycosis diagnosis [6]. Coccidioides meningitis can affect immunocompetent hosts as well. In fact, the count is rising, which has been attributed to climate change and rapid housing development [7–9]. High level of suspicion is necessary to diagnose coccidioides meningitis in immunocompetent patients, and early diagnosis and treatment initiation are imperative to prevent potential life-threatening complication. Therefore, we present a case of coccidioides meningitis in an immunocompetent host without any preceding pulmonary complaints and the diagnostic challenges due to negative serum coccidioides antibody EIA and associated metabolic derangements that resemble coccidioides meningitis.

## 2. Case Report

A previously healthy 38-year-old male presented to the emergency department of a tertiary care center in Nevada during springtime with headaches for five days. His headaches were severe, diffuse, intermittent, and were associated with fever, nausea, nasal congestion, neck stiffness, and photophobia. He had never experienced similar headaches prior to this episode. He immigrated to the United States from India 13 years ago. He is a truck driver, usually drives from Nevada to California and occasionally to Arizona. He denied any other recent travel history, known recent sick contacts, and there were no recent medication changes. He admitted to consuming alcohol and smoking cigarettes about one-pack a day for 10 years, however, denied using any other recreational or intravenous substances.

In the emergency department, he was alert and oriented, afebrile, tachycardic with a heart rate 123 beats per minute, respiratory rate 28 breaths per minute, blood pressure 155/114 mmHg, and oxygen saturation 99% on room air. He had nuchal rigidity on physical examination but no rash or focal neurologic deficit was present. Laboratory findings revealed a white blood cell count 15.2 × 10^3^/μL (normal range 4.8–10.8 × 10^3^/μL) with 83% neutrophils, sodium level 125 mmol/L (normal range 135–145 mmol/L), bicarbonate level 15 mmol/L (normal range 21–32 mmol/L), and lactic acid level 3.7 mmol/L (normal range 0.4–2 mmol/L). A computed tomography (CT) brain without intravenous contrast showed no acute abnormalities. A lumbar puncture (LP) was performed in the emergency department, and CSF analysis is shown below (Table 1).

Additionally, CSF Gram stain revealed many polymorphonuclear leukocytes but no organisms. However, as there were concerns for bacterial meningitis, he was admitted to the medicine floor. Treatment was initiated with intravenous vancomycin, ceftriaxone 2 gm twice a day, acyclovir, and dexamethasone 0.15 mg/kg every 6 h. Our patient initially improved clinically and symptomatically with the antimicrobial regimen. Serum sodium level had improved to 134 mmol/L. A CSF meningitis/encephalitis panel resulted after one day and did not reveal any bacteria, virus, or yeast. Intravenous vancomycin, acyclovir, and dexamethasone were discontinued, and shared decision was made to continue bacterial meningitis treatment with intravenous ceftriaxone 2 gm twice a day for 2 weeks.

However, on Day 3 of hospitalization, the patient reported experiencing mild headaches again. Neurological examination still revealed no focal deficit, and the patient was alert and oriented. Our patient's worsening symptoms prompted a repeat CT brain without contrast and a magnetic resonance imaging (MRI) brain with and without contrast. The CT brain again revealed no intracranial abnormalities. The MRI brain was unremarkable as well with no radiological evidence of meningitis, hydrocephalous, or vasculitis. Serum sodium level on Day 3 was 136 mmol/L. On Day 5, our patient reported worsening headaches, and he had altered mentation on examination. White blood cell count was 16.3 × 10^3^/μL, and serum sodium level was 128 mmol/L on Day 5. He was upgraded to intensive care unit for frequent neurologic examination. Nephrology recommended hypertonic saline for possible acute symptomatic hyponatremia. Lab results as part of hyponatremia workup revealed serum osmolality 274 mOsm/L (normal range 280–300 mOsm/L), urine osmolality 842 mOsm/L (50–1200 mOsm/L, depends on fluid intake and diet), and urine sodium 154 mmol/L (normal range 20–100 mmol/L, depends on fluid intake and diet). Syndrome of inappropriate antidiuretic hormone (SIADH) was suspected, and appropriate management was initiated. Infectious disease reinitiated vancomycin and recommended to obtain serum coccidioides IgG and IgM EIA, and repeat LP with opening pressure, CSF coccidioides antibody, and VDRL screening for syphilis. Tuberculosis and human immunodeficiency virus (HIV) infection were considered as differentials as well prompting screening. However, the screening tests including fourth generation HIV 1&2 Ag/Ab and QuantiFERON TB returned within the normal limit. Repeat CSF analysis on Day 5 is shown below (Table 2).

CSF Gram stain and culture from Day 5 showed no growth either. Serum coccidioides IgG and IgM EIA were still pending. CSF coccidioides serology and antibodies were pending as well. Given suboptimal response to ongoing bacterial meningitis management which was evident by worsening headache and altered mentation, elevated opening pressure, persistent leukocytosis with elevated eosinophils in CSF, concerns were raised regarding fungal meningitis. Fluconazole 400 mg daily was initiated on Day 6. However, serum coccidioides IgG and IgM EIA returned negative on Day 9. Given negative coccidioides EIA and our patient's clinical improvement on bacterial meningitis treatment evident by improved mentation on Day 9, decision was made to discontinue fluconazole. At that time, he was being treated with intravenous vancomycin and ceftriaxone only. He was still experiencing intermittent headaches, and his headaches were thought to be due to sequela from bacterial meningitis. Intravenous dexamethasone was reinitiated which ameliorated the headaches.

Over the next day, he continued to be headache-free. Serum sodium level improved to 135 mmol/L as well. As he improved clinically and symptomatically, shared decision was made to discharge the patient on intravenous ceftriaxone 2 gm twice a day to complete 14-day course for bacterial meningitis and oral methylprednisolone 4 mg 5-day tapered dose for headaches. He was discharged on Day 11, and CSF coccidioides serologies were still pending at the time of discharge. A few days following our patient's discharge, CSF coccidioides IgG and IgM EIA returned positive. CF for CSF titers was less than 1:2, indicating an acute or subacute CNS coccidioidomycosis infection. Attempts to contact him regarding the new diagnosis and change in management were made, however, were unsuccessful.

Our patient returned to the emergency department 1 week after his discharge with recurrence of headaches. He reported completing IV ceftriaxone course as advised. His last dose of methylprednisolone was 2 days before presentation. His headaches recurred the day after he completed methylprednisolone taper and worsened over a span of about 24 h. In the emergency department, he was mildly confused. Nature of the headaches was similar to last episode and was associated with fever, photophobia, and neck stiffness as well. In the emergency department, he was afebrile and tachycardic with a heart rate of 106 beats/minute. Laboratory findings this time were significant for white blood cell count 15.5 × 10^3^/μL. Our patient was informed regarding the coccidioides meningitis diagnosis. He denied any cough, pleuritic chest pain, shortness of breath, joint pain, or rash preceding the headaches. Additionally, a chest imaging over the course of his hospital stay did not reveal any consolidation or nodules suspicious of pulmonary coccidioidomycosis either.

**Figure 1:**
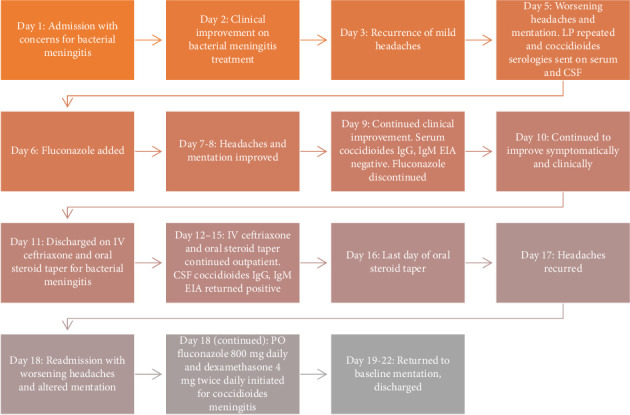
Timeline graph summarizing events.

Treatment was initiated with oral fluconazole 800 mg daily. Oral dexamethasone 4 mg twice a day was started as well to reduce the risk of stroke and other vascular complications. He improved significantly over next few days with complete resolution of headaches and neck stiffness. Shared decision was made to continue fluconazole lifelong for coccidioides meningitis. He was discharged on oral fluconazole 800 mg daily for a few months with plans to taper dose to 400 mg daily depending on clinical improvement at the clinic. Oral dexamethasone was also continued for 4 weeks in a tapering fashion to reduce vasculitic complications. He returned to his baseline functionality with complete symptomatic resolution before discharge.  Figure 1 delineates a timeline graph summarizing hospital admissions, treatment decisions, and serology results.

## 3. Discussion

Valley fever or coccidioidomycosis is a reportable disease in some states of the United States. According to the Centers of Disease Control and Prevention (CDC), a total of 10,000–20,000 cases of Valley fever is reported in the United States yearly. This number could be an underestimation as many cases are not reported, undiagnosed, or misdiagnosed [10]. The annual incidence of Valley fever or coccidioidomycosis is rising as well. Increased housing and human activities in endemic areas have been hypothesized to be linked to the increased incidence rate in endemic regions [8]. Climate change and environmental calamities such as earthquakes and dust storms facilitate dispersion of arthroconidia. Also, cases reported in nonendemic states had shone light on the possibility that the geographic range of the organism is likely larger than it was previously thought [9]. Certain occupation including long-haul driving, construction, farm work, and excavation increase the risk of arthroconidia exposure as well.

Coccidioidomycosis can be complicated by dissemination. Patients more than 65 years old; with uncontrolled diabetes, smoker, pregnancy, and depressed cellular immune system such as HIV infection; or organ transplant recipient are high risk for developing disseminated disease [11, 12]. CNS involvement can occur in up to half of patients with disseminated coccidioidomycosis; CNS symptoms usually manifest within weeks to months of primary infection [13]. Headache is usually the most common symptom, altered mental status, nausea, vomiting, and focal neurologic deficits, with or without fever may be present as well [14, 15]. LP with CSF analysis is recommended in patients with recently diagnosed coccidioides infection if they develop symptoms concerning meningitis [16].

CSF analysis shows pleocytosis, usually lymphocytic, however, can also be neutrophilic during early disease [14, 17]. CSF opening pressure is usually elevated. Other significant CSF findings are low glucose and elevated protein [15–17]. Elevated CSF eosinophil or eosinophilic pleocytosis has been historically associated with coccidioides meningitis [18]. Positive CSF serology for IgG antibody is considered diagnostic for coccidioides meningitis [19]. A retrospective analysis on coccidioides meningitis conducted in a referral center in Central Valley, California, from 2010 to 2020 detected positive CSF coccidioides IgG in 86% of the patients and serum coccidioides IgG in 94% of the patients [2]. CF measures the IgG antibody titers, and maximal titer takes a mean of 31 days to develop [19]. However, not all patients will develop coccidioidal CF titers. ID can detect IgM in 1–3 weeks, followed shortly by detection of IgG. ID can be used to diagnose coccidioides meningitis in case CF fails to detect antibody titers [19]. Fungal culture and direct microscopy are less common but are diagnostic if positive.

Despite recent advancements in diagnostic approaches, diagnosis of coccidioides meningitis is still time-consuming. Different disease categories of coccidioidomycosis have been shown to have a median diagnostic delay ranging from 17–54 days in 2019 [20]. Average delay for coccidioides meningitis had been shown to be as high as seven months in 1970 [21]. No recent data are available on the average delay in diagnosing coccidioides meningitis. Delayed diagnosis is likely due to its association with nonspecific symptoms and time required for the antibodies to become detectable as evident in our case. The equivocal presentation of our patient and initial neutrophilic pleocytosis mimicked bacterial meningitis which had led to initiation of antibacterial in an appropriate clinical setting. As our patient's symptoms recurred after initial improvement, neuroimaging with repeat CT brain and MRI brain were obtained. Neuroimaging, especially MRI brain, has been shown to be useful in the diagnosis of coccidioides meningitis [14]. Neuroimaging can reveal meningeal involvement, hydrocephalous, or vasculitic complications. However, neuroimaging reveals hydrocephalous in only half of the patients and vasculitic complications in about one-fifth of the patients [14]. Likewise, in our patient, neuroimaging was undiagnostic of any intracranial process.

Metabolic and electrolyte derangement can clinically resemble coccidioides meningitis as well. In our patient's case, recurrence of headaches and altered mentation were thought to be related to hyponatremia. Hyponatremia has rarely been reported as a complication of coccidioides meningitis, likely due to SIADH [22]. To the authors' best knowledge, only two case reports had depicted SIADH as a vasculitic complication of coccidioides meningitis [22, 23]. Our patient's urine studies were also significant for SIADH, and in his case, SIADH was thought to be a complication of coccidioides meningitis as well. The presence of SIADH in our patient posed a unique diagnostic challenge as both hyponatremia and coccidioides meningitis share similar symptomatology, further attributing to delayed diagnosis of coccidioides meningitis.

Our patient had environmental exposure given his occupation. However, he had no pulmonary symptoms of coccidioidomycosis. Pulmonary symptoms can be absent in about half to one-third of the patients. Nonetheless, in our case, CSF eosinophilic pleocytosis along with elevated CSF opening pressure raised the suspicion of coccidioides meningitis. His serum anti-coccidioides IgG and IgM EIA were negative. This was likely due to the fact that he was still in early phases of infection for IgM to form, and formation of IgG antibody takes even longer. We used CSF coccidioides IgG and IgM EIA to guide the diagnosis and positive CF assisted to further solidify our suspicion. However, CF titers were low in our case likely because our patient's possible exposure was within less than 2-3 weeks. ID was inconclusive in our case, likely due to recent exposure and early disease phase. High dose fluconazole was initiated on our patient with plans to continue lifelong. Lifelong azole treatment has been recommended for coccidioides meningitis [16]. Initiation of azole provided symptomatic relief to the patient and helped him to return to his baseline functionality. Empiric treatment with high dose fluconazole has been recommended for suspected cases of coccidioides meningitis even with negative serologies, particularly in immunocompromised patients.

We present this case to elaborate the diagnostic challenges of coccidioides meningitis. Early diagnosis and treatment of coccidioides meningitis is imperative to prevent long-term cerebrovascular complications, and the key to early diagnosis is keeping a high index of suspicion. Unfortunately, serology can be undiagnostic or inadequately diagnostic during early phases of the disease. In our case, CSF serologies were indicative of CNS coccidioidomycosis, even though the titers were low. But, in cases with inconclusive or inadequate serologies, it would be appropriate to initiate antifungal to prevent long-term complications of coccidioides meningitis. Also, as evident in our case, SIADH is a rare metabolic derangement associated with coccidioides meningitis. Unexplained hyponatremia in patients with coccidioides meningitis could be associated with SIADH. This association has been very rarely reported and can institute further diagnostic challenges as portrayed in our case.

## 4. Conclusion

Regardless the recent enhancements in the diagnostics, it is still challenging to establish a definitive diagnosis for coccidioides meningitis. So it is crucial for the physicians to have high level of suspicion for coccidioides meningitis, even in immunocompetent patients, given the rising incidence of the disease and its unforgiving sequela.

## Figures and Tables

**Table 1:** CSF analysis on Day 1.

Test	Results	Units	Reference
CSF appearance	Clear		Clear
CSF color	Colorless		Colorless
White blood cell	2.5	×10^3^ μL	0.000–0.005
Red blood cell	< 0.002	×10^6^ μL	0.000
CSF polynuclear WBC	67	%	0
CSF lymphocytes	20	%	40–80
CSF eosinophils	3	%	0
CSF glucose	24	Mg/dL	45–70
CSF total protein	173	Mg/dL	15–45

**Table 2:** CSF analysis on Day 5.

Test	Results	Units	Reference
CSF appearance	Clear		Clear
CSF color	Colorless		Colorless
CSF opening pressure	37	cmH_2_O	10–20
White blood cell	0.4	×10^3^ μL	0.000–0.005
Red blood cell	< 0.002	×10^6^ μl	0.000
CSF polynuclear WBC	4	%	0
CSF lymphocytes	80	%	40–80
CSF eosinophils	6	%	0
CSF glucose	17	Mg/dL	45–70
CSF total protein	92	Mg/dL	15–45
CSF VDRL	Non-reactive		Non-reactive
